# Unmanned Surface Vehicle Simulator with Realistic Environmental Disturbances

**DOI:** 10.3390/s19051068

**Published:** 2019-03-02

**Authors:** Marcelo Paravisi, Davi H. Santos, Vitor Jorge, Guilherme Heck, Luiz Marcos Gonçalves, Alexandre Amory

**Affiliations:** 1 Instituto Federal de Educação, Ciência e Tecnologia do Rio Grande do Sul, Osório RS 95520-000, Brazil; marcelo.paravisi@acad.pucrs.br; 2 Pontíficia Universidade Católica, Porto Alegre RS 90619-900, Brazil; vitormj@ita.br (V.J.); guilherme.heck@acad.pucrs.br (G.H.); 3 Universidade Federal do Rio Grande do Norte, Natal RN 59078-970, Brazil; davihenriqueds@gmail.com (D.H.S.); lmarcos@dca.ufrn.br (L.M.G.); 4 Electronics Engineering Division, Instituto Tecnológico de Aeronáutica, São José dos Campos SP 12228-900, Brazil

**Keywords:** robotics simulation, unmanned surface vehicles, computational fluid dynamics, hydrological modelling, bridge inspection

## Abstract

The use of robotics in disaster scenarios has become a reality. However, an Unmanned Surface Vehicle (USV) needs a robust navigation strategy to face unpredictable environmental forces such as waves, wind, and water current. A starting step toward this goal is to have a programming environment with realistic USV models where designers can assess their control strategies under different degrees of environmental disturbances. This paper presents a simulation environment integrated with robotic middleware which models the forces that act on a USV in a disaster scenario. Results show that these environmental forces affect the USV’s trajectories negatively, indicating the need for more research on USV control strategies considering harsh environmental conditions. Evaluation scenarios were presented to highlight specific features of the simulator, including a bridge inspection scenario with fast water current and winds.

## 1. Introduction

Unmanned Surface Vehicles (USV) are now being used in applications such as search and rescue, containment of oil spills, structural inspection of bridges, tsunami/earthquake forecast, homeland security, and environmental monitoring, among other applications [1,2,3]. USVs offer significant advantages over other robotic platforms (aerial and underwater), including payload and energy capacities [4], localization resources [5], as well as access to conventional data communication capabilities [6].

In spite of their importance in numerous tasks, this paper shows that the ability to simulate USVs remains rare in popular robotics simulation frameworks. One of the main challenges of such simulators is the requirement to tackle complex environmental disturbances such as waves, winds and water currents [7]. The lack of a standard simulation environment has adverse effects on USV research. Among them, we highlight the absence of a standard modular testing platform for USV Guidance, Navigation and Control (GNC), which makes it challenging to perform comparisons and benchmark these GNC methods. The difference in the way natural disturbances are modeled has important effects on GNC strategies, especially in small USVs due to their low inertia and small size [8]. A GNC strategy which does not address environmental disturbances is likely to perform poorly in the field without ideal weather conditions, especially in unpredictable disaster scenarios.

Towards solving this problem, and expecting to assist USV control designers and USV researchers to benchmark their approaches, this paper presents an open-source 6-Degree-Of-Freedom simulator for USVs, integrated with the Robot Operating System (ROS)-based framework, which models environmental disturbances like winds, water currents, and waves. The proposed tool can simulate different boats, such as those with differential propellers, a single propeller and rudder, airboats/hovercrafts, and sailboats.

The main contributions of this research work are:

- a freely available modular USV simulator where it is possible to model different boats, evaluation scenarios, and GNC strategies. The source code of the simulator, USVsim, is available [9] with an open-source license;
- improvement of buoyancy effects, influencing USVs’ roll, pitch, and yaw;
- integration of a wind model which affects parts of the boat above the line of water;
- integration of a hydrological model to simulate water currents of water bodies, applying forces to the boat;
- ready-to-use USV models and simulation scenarios;

This paper is organized as follows. Section 2 reviews open source simulators related with USV. Section 3 presents the proposed USV simulator and the USV simulation models. Section 4 shows how two USV models were validated against two real USVs. Section 5 details the evaluation scenarios and results. Section 6 concludes this paper.

## 2. Related Work

There are surveys in the literature on the topic of computer-based simulators for unmanned systems. Craighead et al. [10] propose four evaluation criteria: physical fidelity, functional fidelity, ease of development, and cost. The authors conclude that the development of a new simulator, from scratch, may no longer be needed. Instead, research should focus on improvements over existing simulators, which should be shared and released to the Robotics and Automation (R&A) community. Still, in similar work, Harris and Conrad [11] add that the diversity of simulated sensors, the accuracy of graphics and physics, cross-platform capabilities, and also the availability of source code are also important evaluation parameters. Torriti et al. [12] survey only free simulators and they concluded that the number of simulators supporting USVs is reduced when compared to other platforms.

Initially, in order to check this last claim, ten open source simulators (Kelpie [13], USARSim [14], MARS [15], Stage [16], MOOS-IvP [17], UW-Morse [18], UWSim [19], Gazebo [20], “FreeFloating” plugin [21], V-REP [22], RobotX Simulator [23] were evaluated related to their USV simulation capabilities. We discarded from the comparison simulators whose code was not available (Kelpie, UW-Morse), discontinued simulators (MARS) or those which were difficult to compile due to outdated libraries (USARSim). Simulators without built-in physics capability, such as Stage and MOOS-IVP, were also excluded since built-in physics is a minimal requirement to simulate disturbances of any kind (gravity, environmental, collision, among others). Lastly, in Table 1, the remaining four simulators (UWSim [19], Gazebo [20], “FreeFloating” plugin [21], and V-REP [22]) were classified according to the following features:

- Waves: indicates the specific ability to simulate 3D waves and offers ways for integration with vehicle simulation (√√), i.e., there are waves simulations, but they are for visualization purposes only (√), or inability (×), to visually simulate waves;
- Buoyancy: Archimedes’ principle which makes the USV float over waves. This criterion has been classified into three levels: no buoyancy (×), which means the robot moves over a rigid plane and waves have no influence on the boat; simplified buoyancy (√), which means it only performs rigid vertical movement of the entire vessel, according to the height of the water plane; improved buoyancy (√√), where the boat tends to follow the shape of the water, resulting in pitch and roll rotations;
- Water currents: describes the method used to apply the water current forces to the vessels: not applied (×); a constant force over the time and space (√); or a variable force over the time and space (√√), according to some Computational Fluid Dynamics (CFD) model;
- Wind currents: similar to the previous item, but the wind force is applied only for objects above the line of water;
- Underwater thruster: how the thruster force is simulated under the water: not simulated (×); a linear function computes the percentage of a maximum force to be applied by the thruster (√); simulates the dynamics effects of the helix blades of the propeller (√√);
- Above water thruster: the same as the previous item, but this thruster is used above the surface of the water, such as in airboats;
- Foil: how the foil dynamics are simulated: not simulated (×); simulates nonlinear Lift and Drag forces on the foil (√√).

UWSim [19] is a modular and extensible tool on underwater simulations and Unmanned Underwater Vehicles (UUVs). It offers visually realistic wave and underwater simulations, working mainly as a visualization tool for external modules such as Gazebo, which is responsible for the control algorithms, vehicles, and environment dynamics. It provides ready-to-use scenarios and one UUV named Girona 500 UUV, with the ARM5 manipulator. Its main limitations for boat simulation include: lack of dynamics of a rudder; simplified water current models (i.e., a single constant force is applied to the entire scenario); the wind simulation appears to only affect the wave shape and height, not the vehicle movement; and finally it is not straightforward to model realistic boat behavior, since its focus is clearly on UUVs.

Gazebo [20] is a popular simulation tool for unmanned systems. It can simulate different robots and complex 3D environments together with the support of several physical simulation engines. Its modular structure enables the extension of its core features through plugins. Most of the current development efforts using Gazebo focus on Unmanned Ground Vehicles (UGVs) and UUVs. Few works introduce new features designed for USVs, such as the RobotX Simulator and the FreeFloating plugin.

The development of RobotX Simulator started in the early 2018, and its primary objective is to host the Virtual Maritime RobotX Competition (VMRC). The VMRC aims to be the entry point for teams that aspire to participate in the Maritime RobotX Challenge [24]. The simulator builds upon the ROS/Gazebo environment and is under development. It currently provides a model for the WAM-V, a catamaran with differential actuation used for the RobotX competition. This virtual model presents the effects of buoyancy, waves, and also some hydrodynamic effects such as added mass and damping. It also has a simplified wind model (a constant force). The simulator does not provide modules for foil dynamics or realistic wind and water currents. Although the thrusters can be simulated by a linear function of maximum force (defined by the user), this feature can be upgraded by using the LiftDrag plugin of Gazebo Simulator.

FreeFloating [21] is a simulation tool directed for underwater robots used in conjunction with UWSim and Gazebo. It offers a simple buoyancy scheme (applied to the submarine, until it reaches the surface plane Z = 0) and the viscous force of water over UUVs while at the same time enabling the control over wrench, joint states, and body velocity of robots. It can also be combined with the LiftDrag [25] Gazebo plugin to simulate the aerodynamics of thruster blades. However, even when combined with all those modules (see Table 1), it does not fully implement rudder models, airboats, or even realistic wind or water currents.

V-REP [22] supports add-ons, plugins, socket communication, ROS integration, and Lua scripting. It can use particle simulation to emulate air or water jets, propellers and jet engines, even though, considering the demos and the documentation available on the website, there is no working boat usage example. There is a differential USV model implemented into V-REP (GAMS [26]) with a simple visual effect of waves (no effect on the boat’s pose) and the vehicle floats over a flat surface. As far as we know, there is no simulation of the influence of water and wind currents on the boat, or a readily available model to describe the rudder dynamics on V-REP.

This study of reviewing existing simulation environments for USV revealed the best features and limitations of each of them. Following the conclusions of [10], we decided to build an improved simulator reusing the following tools:

- Gazebo: as the core simulation engine, due to its modular design based on plugins, dynamics & collision simulation, number of modeled sensors, USVs can be designed using XML-based URDF/Xacro format, and the ease of development given the large community of users and code maintainers;
- Free-floating Plugin: Despite some limitations detailed later, the structure of this plugin, originally developed for underwater robots, was re-purposed to be used for USV simulation and we improved the hydrodynamics and buoyancy effects compared to the original plugin;
- UWSim: for water and wave visual effects and due to sensors that are readily available for use;
- LiftDrag Gazebo Plugin: we mainly reuse its structure to calculate foil dynamics in the parts of the boats.

These original plugins and simulators where improved and re-purposed for use in USV simulation and their improvements are shown in the following section.

## 3. System Architecture

**Figure 1:**
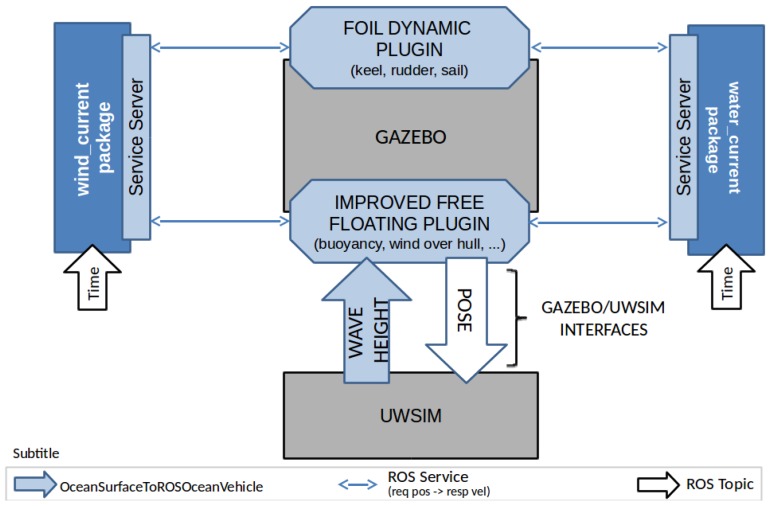
Overall simulation architecture and its main modules.

This section presents the proposed simulation architecture and the main contributions designed on top of the available simulation resources presented in Section 2. Figure 1 gives an overall description of the system architecture where the blue boxes represent the new or customized simulation modules.

Gazebo is used as the main simulation engine while UWSim is used for visualization purposes. As represented in Figure 1, the core of Gazebo is not modified, but we included one new plugin called usv_foil_dynamics and also the improved FreeFloating. UWSim has been modified to provide a service where the plugins request the wave height at a specific position of the map. The water and wind current generators are modeled as ROS nodes which receive requests from improved FreeFloating Gazebo to enhance the boat motion realism through wind and current information. Besides, the wind/water current generator is used by the foil dynamics plugin to compute the forces which are directly applied to the foil (if the boat has one), using as input the velocities of the foil and the velocity of the wind/water current. All modules have their corresponding YAML files where the user can set specific parameters without changing the source code.

The rest of this section details each relevant module and it is organized as follows. Section 3.1 introduces the theoretical aspects of the underlying mathematical models. Then, Section 3.2 details the Foil Plugin, used to simulate the boat’s rudder, keel, and sail. Section 3.3 describes the main improvements made in the existing simulators. Section 3.4 describes the four ready-to-use USV models provided with the simulator. Finally, Section 3.5 presents the Computational Fluid Dynamics (CFD) tools integrated to the simulator to address water and wind models.

### 3.1. Introduction to the Main Forces for a USV

According to Fossen [27] the main forces that govern a USV movement are:

- Hydrodynamic forces ($\tau_{\mathrm{hyd}}$): added mass (the virtual mass added to the boat by the mass of water moved with the boat), potential damping and viscous damping;
- Hydrostatic forces ($\tau_{\mathrm{hs}}$): restoring forces (buoyancy);
- Wind forces ($\tau_{\mathrm{wind}}$);
- Wave forces ($\tau_{\mathrm{waves}}$);
- Control and propulsion forces (τ): foil dynamics (rudder, keel and sail) and thrusters;

The resultant USV movement is given by the combined effect of those forces and moments, as shown in Equation (1).

(1) $$\tau_{\mathrm{RB}}=\tau_{\mathrm{hyd}}+\tau_{\mathrm{hs}}+\tau_{\mathrm{waves}}+\tau_{\mathrm{wind}}+\tau$$

In the simulator presented in this paper, these forces are calculated employing Gazebo plugins and applied to the USVs by the Gazebo Engine, with each plugin being responsible for computing one or more forces. This allows the user to control the level of fidelity/performance they need for their application. The Hydrodynamic forces ($\tau_{\mathrm{hyd}}$), Hydrostatic forces ($\tau_{\mathrm{hs}}$), Wind forces ($\tau_{\mathrm{wind}}$), Wave forces ($\tau_{\mathrm{waves}}$) and Thruster dynamics (part of τ) are implemented by an improved Free-Floating Plugin. The Foil Dynamics Plugin performs the Control and propulsion forces (τ), namely the Foil Dynamics. Details of these plugins are presented next.

### 3.2. Foil Dynamics Plugin

The Foil Dynamics Plugin is used to calculate the Lift and Drag forces (τ) that act on the foils of the boat, namely the rudder, keel and/or sail, depending on the type of boat. To estimate those forces, it calculates the apparent velocity between the foil and the fluid (water or air, depending on the foil) and it uses the specific drag and lift coefficients of the foil to compute the lift and drag forces applied, according to the following equation:

(2) $$\left[\begin{array}{c} F_{x}\\ F_{y} \end{array}\right]=\left[\begin{array}{c} \frac{1}{2}\rho A\upsilon_{a}^{2}C_{L}\left(\alpha \right)\sin\alpha -\frac{1}{2}\rho A\upsilon_{a}^{2}C_{D}\left(\alpha \right)\cos\alpha \\ \frac{1}{2}\rho Av_{a}^{2}C_{L}\left(\alpha \right)\cos\alpha +\frac{1}{2}\rho A\upsilon_{a}^{2}C_{D}\left(\alpha \right)\sin\alpha \end{array}\right],$$

where $F_{x}$ is the drag, $F_{y}$ is the lift, ρ is the fluid density, *A* is the area of the foil, $\upsilon_{a}$ is the apparent speed of the fluid, α is the angle of attack of the foil. $C_{L}\left(\alpha \right)$ and $C_{D}\left(\alpha \right)$ are the lift and drag coefficients respectively, as functions of the angle of attack.

**Figure 2:**
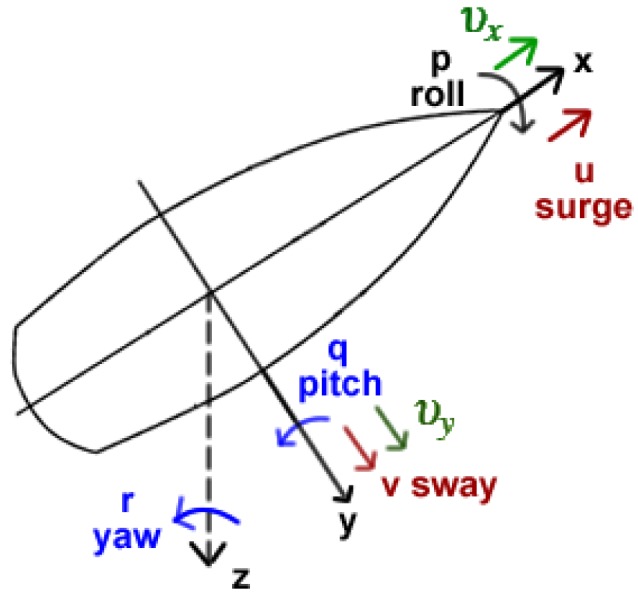
Boat coordinate frame ($\upsilon_{x},\upsilon_{y}$) and the apparent water velocity components.

The apparent fluid speed can be computed as $\upsilon_{a}=\sqrt{\upsilon_{x}^{2}+\upsilon_{y}^{2}}$, where $\upsilon_{x}$ and $\upsilon_{y}$ are the apparent fluid velocity components along the longitudinal and lateral axes of the boat reference frame (see Figure 2), which are estimated as

(3) $$\begin{array}{c} \upsilon_{x}=\upsilon_{t}\cos\left(\alpha_{t}-\psi \right)-u+rn\\ \upsilon_{y}=\upsilon_{t}\sin\left(\alpha_{t}-\psi \right)\cos\phi -v+rm+po \end{array}$$

where $\upsilon_{t}$ is the true fluid speed, $\alpha_{t}$ is the angle of true fluid, ψ is the boat yaw angle, and the components of the boat velocity vector are ${\left[u,v,p,r\right]}^{T}$, i.e., surge, sway, roll and yaw respectively. The Center of Effort of the foil is at position (m,n,o) in the boat reference frame. For example, for a rudder the angle of attack $\alpha_{r}$ can be computed by $\alpha_{r}=\alpha_{aw}-\delta_{r}$, where $\delta_{r}$ is the rudder angle in the boat reference frame and the angle of the apparent water current is given by $\alpha_{aw}=a\tan2\left(\upsilon_{y},-\upsilon_{x}\right)$.

While the original LiftDrag plugin computes the forces only considering the foil velocity, the proposed plugin considers the speed of the fluid and the foil to calculate the lift and drag forces. Besides, the Foil Dynamics Plugin uses the values of Lift and Drag coefficients described in [28], which approximates the coefficients by using $C_{D}\left(\alpha_{a}\right)=\sin\left(2\alpha_{a}\right)$ and $C_{L}\left(\alpha \right)=1.5\left(1-\cos\left(2\alpha_{a}\right)\right)$, where $\alpha_{a}$ is the apparent angle of attack. In this way, the user only needs to inform the physical characteristics of the foil, i.e., area, frontal and side directions, and a central point to apply the lift and drag forces.

### 3.3. Improvements on UWSim and Free Floating Gazebo Plugin

**Figure 3:**
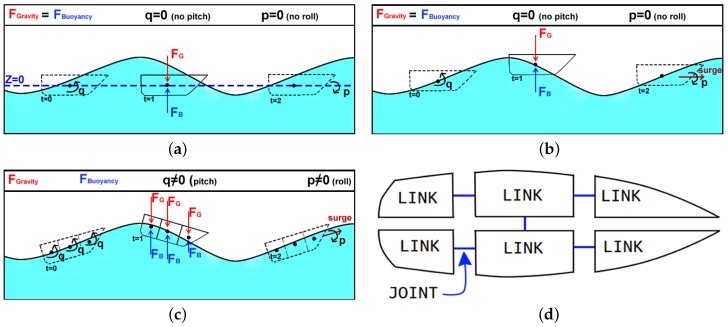
Different implementations of the buoyancy effect are shown in (**a**–**c**), where black dots represent the center of buoyancy, while red (downward) and blue (upward) arrows represent the gravity and buoyancy force vectors respectively. In (**a**), the buoyancy effect of Freefloating gazebo is shown [21]. In (**b**), we present the buoyancy effect on a boat which takes the wave height into account, given the boat is defined by only one link. In (**c**), a similar effect is shown but the boat is now represented by a set of links and joints subdivided as in (**d**). Note that the gravity and buoyancy forces are applied to each of the links’ center of buoyancy.

Gazebo currently does not simulate water current and waves. In UWSim, on the other hand, waves are rendered by a library called OsgOcean, which implements wave behavior using the Fast Fourier Transforms. Although UWSim knows the height of waves, this information is not shared with Freefloating Gazebo [21]. So all boats float on a flat water surface plane (Z = 0), ignoring the shape of waves and restraining the simulation realism as shown in Figure 3a.

For this reason, we modified UWSim by synchronizing its simulation of waves (osgOcean) with Gazebo through a new interface called OceanSurfaceToROSOceanVehicle. Such modification ensures that the simulation step of osgOcean will be the same for all Gazebo plugins. When a vehicle’s pose is updated in UWSim, the wave height relative to the vehicle’s center(s) of buoyancy is sent through this interface to our improved Freefloating Gazebo, which is used as input for the buoyancy effect. Although this allows boats to float up and down with the wave motion, sometimes it causes the front (bow) and the rear (stern) of boats to be either floating outside the water or fully submerged, as illustrated in Figure 3b.

Therefore, we further modified Freefloating Gazebo in order to allow boats to have roll and pitch rotations caused by wave motion ($\tau_{\mathrm{waves}}$), allowing the boats to follow the slope of waves as illustrated in Figure 3c. In such strategy, each boat hull must be subdivided into several parts, i.e., modeled by a set of links bounded together by fixed joints (see Figure 3d). This subdivision also improves the inertial momentum of the boat’s hull since each part may be in different wave heights, submerged hull volume, and buoyancy forces. These individual forces under each part of the hull are combined by the Gazebo’s physics engine, allowing the torque to be estimated. The resulting effect of the gravity (red arrow) and buoyancy (blue arrow) forces is depicted in Figure 3c, allowing the boat to roll and pitch due to the influence of waves, causing a more realistic wave effect on the boat’s movement. As the boat hull is defined by a set of links, we can reduce the complexity of submerged volume by representing each link by a primitive geometry (cubes, cylinders, etc.), thus we can avoid computationally expensive methods that can restrict the simulation speed.

In addition, we customized Freefloating Gazebo to allow wind forces to affect boat hulls and their motion ($\tau_{\mathrm{wind}}$). So, the boats presented next, named Airboat, Rudder Boat, Differential Boat, and a Sailboat can also be affected by the wind forces applied into the hull, while the sailboat is affected by the wind forces applied into the sail and the hull. We apply the wind forces and moments to each link of the boat’s hull, but we consider the aerodynamic coefficients of lateral and longitudinal resistances, as well as the cross-force and rolling moments proposed by [29].

### 3.4. Proposed Robot Models

**Figure 4:**
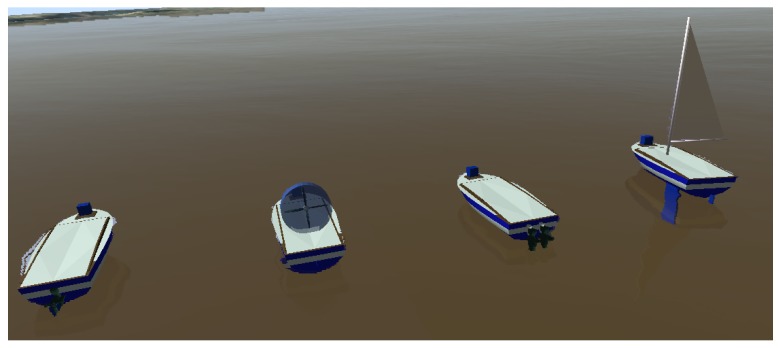
From left to right: the rudder boat, airboat, differential boat and sailboat available in the simulator.

Four different ready-to-use USV models, illustrated in Figure 4, are integrated into the proposed simulator as default in order to describe different dynamics of USV: an airboat, a motorized boat with one rudder, a differential boat with two thrusters, and a sailboat. The airboat uses a fan above the surface of the water as a propeller. This fan also rotates on its own axis to change the airboat direction of movement. The airboat suffers more from the effect of drift (sideslip angle) since it does not have any foil underwater. The motorized boat uses an underwater thruster for propulsion and a rudder for changing the movement direction. The differential boat uses two underwater thrusters, which enables the rotation over its axis. The sailboat uses a rigid sail for propulsion, a rudder for changing the movement direction, and it also has a keel to help with sway and roll stability.

These boats can have a variety of sensors thanks to Gazebo, which also offers large documentation on how to model personalized sensors such as water current, temperature, pH, etc. Currently, the ready-to-use USV models have position sensors, Inertial Measurement Unit (IMU) and a laser range finder.

Regarding usage for disaster applications, the Airboat is best used in shallow waters, with underwater debris that could damage rudders or thrusters, and areas with eelgrass that could block the underwater thrusters. However, the Airboat is not recommended for places with strong currents and wind. In addition, it has reduced payload capacity compared to the other boats. The differential boat is one of the most versatile boats due to its increased maneuverability and power to withstanding currents. Although it also consumes more energy due to the twin motors, reducing the time of the mission. The rudder boat is a compromise between energy consumption, maneuverability, and payload capacity. The sailboat is best used in large water bodies or at sea due to its reduced power consumption, able to withstand long missions. The sailboat cannot be used in shallow waters due to the size of the keel.

The dimensions of the airboat and differential boats are presented in Section 4.1, according to the dimensions of two real boats available at the laboratory. The rudder boat uses the same hull and the same thruster used by the differential boat, and it has a rudder of 4 centimeters wide and 6 centimeters tall. The sailboat also uses the same hull, and it has a sail of 1 m^2^ area and a keel of 30 Kg.

### 3.5. Water and Wind Current Modules

An actual scenario is used as study case for this paper. This place is located at 30°02′50.5″ S 51°13′57.7″ W, in the city of Porto Alegre, Brazil, nearby the Dilúvio’s river mouth. This scenario is built based on digital terrain models provided by the Municipality of Porto Alegre.

**Figure 5:**
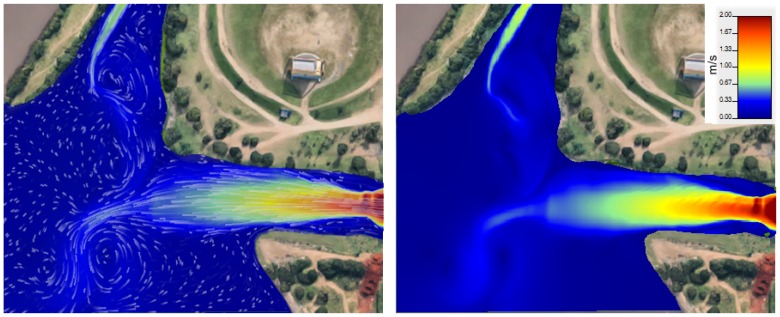
Hec RAS simulation of water current for a given environment showing the intersection of two rivers. The image on the left shows white particle trails moving on the water. On the right, the fastest flow is depicted in red, while the slowest is in blue.

In order to allow water currents to change across space and time more realistically we developed a new ROS package called water_current, which loads data exported from the Hec RAS [30] hydrological simulator [31]. Hec RAS can model one-dimensional (1D) steady flow, as well as one and two-dimensional unsteady hydrologically-based flow calculations in rivers and canals. Thus users can simulate the flow of rivers by inserting simple height maps from terrain and river bed, then exporting Hierarchical Data Format (HDF) files, which store the water velocities for each time step of the simulated water flow. Figure 5 shows the resulting water current velocity map, where the water flows to the left-hand side of the image. Note that the narrow channels have higher speeds. By using the HDF files as input for water_current plugin, our simulation architecture requests the velocity of the water at each of the boat’s position of links. This is done by a ROS service, where the improved FreeFloating Gazebo sends the (x,y) position of each link (see Figure 3d) and the water_current answers with a flow velocity for each point.

Similarly, the wind_current ROS package loads wind data exported by the CFD software OpenFoam [32] (Open source Field Operation And Manipulation). Openfoam is composed of a C++ library capable of solving specific continuum mechanics problems and it provides several utilities to prepare a mesh for simulation, process results, and so on. It generates wind simulation based on a 3D terrain model with multiple obstacles, such as buildings and bridges, and by specifying the simulation parameters, like wind velocity, simulation duration, and time step.

**Figure 6:**
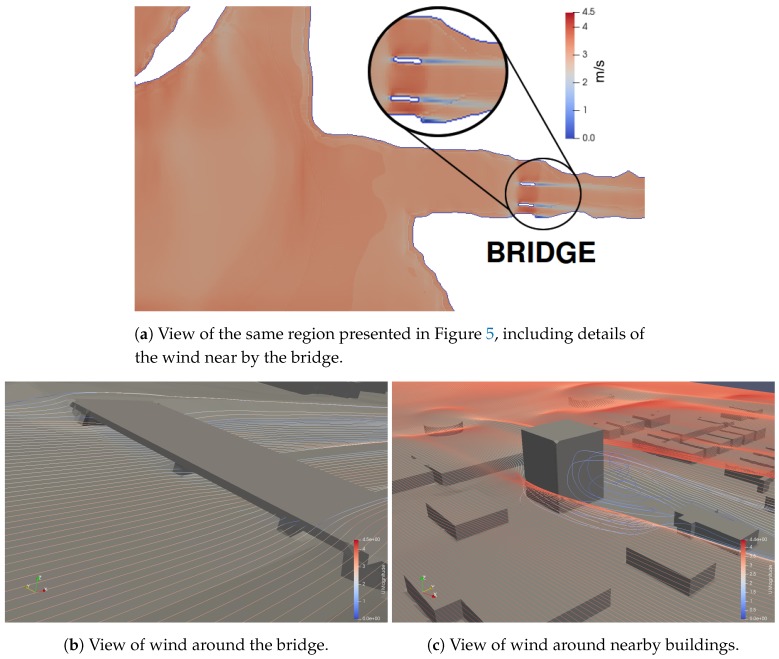
Wind simulation within OpenFoam.

The resulting simulation can be analyzed and exported to the wind_current module by an OpenFoam utility named paraView. The data exported by paraView contains the wind velocity at each time step, that can be loaded into the wind_current module and accessed in the simulation architecture by a ROS service. To do that, the improved FreeFloating Gazebo sends the vehicle’s position of each link, the wind_current package answers with wind velocity for each point. Figure 6 shows the resulting wind model for the proposed test scenario, including examples of wind models around the bridge and buildings.

## 4. Field Trials Validation

In order to verify the fidelity of the temporal responses in the simulator, the airboat and differential boat were modeled in simulation according to two existing physical boats that we have available in our laboratories.

### 4.1. Physical Boats Specification

The airboat and differential boats were modeled according to specifications of the Lutra Airboat and Lutra Prop boats, respectively, acquired from Platypus [33]. Lutra Airboat and Lutra Prop boats have the same hull dimensions and shape, both are 106 centimeters long, 48 centimeters wide and 15 centimeters tall. While Lutra Airboat weights 9 Kg, Lutra Prop weights 9.7 Kg. In our trails, both boats carried approximately 3 Kg of extra payload, which was needed to properly transport a Real Time Kinematic (RTK) system (RTK antenna, RTK module, power bank, antenna pole, cellphone with internet access). Lutra Airboat is driven by a propeller coupled to the hull’s stern, providing forces parallel to the boat’s hull. On the other hand, the Lutra Prop boat has two underwater propellers, both are attached such that there is an angle of 15 degrees between the horizontal line and the propeller’s axis.

The main physical specifications of both boats are presented in Table 2. Most of them are collected by laboratory measurements. Thruster force, linear drag coefficient and maximum speed are estimated by field trials as described in Section 4.2.

### 4.2. Parameters Extraction Procedure

To obtain simulation results such as those observed in the real world, the virtual boats should be configured with physical parameters of the real boats. To do that, the methodology used for collecting such physical data is based on the work of Wirtensohn [34], where the technical data are: length, width, mass, additional payload, electric motor power, and supply voltage level. Other parameters like damping coefficients and maximum speed are acquired by executing experiments in the real world. The robot is tied to a pole connected to a dynamometer. Then, to identify the thrust force, different speed values are applied to the boat, and the resulting force is measured using the dynamometer. Also, to identify surge dynamics, the robot is accelerated to the maximum speed followed by a sudden decrease of the thruster force to zero. Since the resulting deceleration of the boat is caused only by damping, the damping coefficients can be estimated.

We use a similar procedure to identify the thruster force and surge dynamics of both Lutra Airboat and Lutra Prop. However, since the input data of the thrusters is expressed in PWM values, we modify the Wirtensohn’s strategy in order to use PWM values instead of speed for obtaining the maximum of thrust force. Several PWM values have been applied to the actuators and the force value is measured by a dynamometer considering the force value obtained after the force measure stabilizes.

On the Lutra Airboat, we measure the thrust force by applying the following Pulse Width Modulation (PWM) values: 2000, 1850, 1750, 1650, and 1500. The results presented in Table 3 show a linear growth in the force power, while the value of 1500 is insufficient to activate the propeller. On the Lutra Prop, we applied the following PWM values: 1800, 1700, 1600, 1553 and 1500. Similar to the Airboat, the propeller is not activated when 1500 is applied. The maximum thrust force value that we manage to measure is 1700 since the PWM value 1800 has caused the shutdown of the electric system once a fuse blew up because the motors drained current beyond the safe limits of the electrical system.

### 4.3. Comparison between Physical and Simulated Boats

Each boat starts still and then we put the maximum PWM values mentioned before (2000 for Airboat and 1700 for Differential), to move the boats straight ahead. This enables us to obtain the velocity time response of both simulated and real boat, as presented next.

The *physical boats* were equipped with Emlid’s Reach RTK Global Positioning System (GPS) [35]. The GPS was configured to received the corrections from a public base station provided by IBGE [36], located at 30°04′26.7″ S 51°07′11.2″ W, via the NTRIP protocol (Networked Transport of Radio technical commission for marine service—RTCM—via Internet Protocol). The lake used for the test is located at 30°04′57.9″ S 51°03′23.9″ W, and it has an area of about 85 m by 55 m. The distance from the base station to the lake is about 6.1 km, guaranteeing optimal GPS corrections. During the tests, the GPS got more than 99% of fix (best GPS signal), generating excellent accuracy. Finally, there was no significant wind at the test location during the tests. All these characteristics of the test location and test moment were selected to increase the localization accuracy of the physical boats. The *simulated boats*, on the other hand, have the advantage of a perfect localization, easing the velocity analysis presented next.

**Figure 7:**
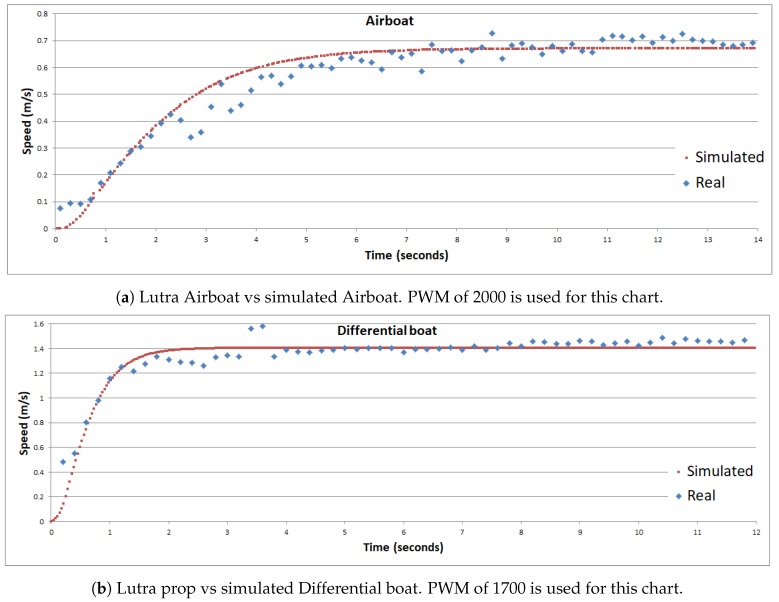
Comparison of speed trials between physical and simulated boats.

Figure 7 shows the comparison of the speed profile obtained in the field trial and simulation for both the Airboat and the Differential boat. Both charts show speed (m/s) per time (in seconds), assuming that time zero was the moment the boats started to move. However, the physical boats have a starting velocity close to zero due to no significant wind and water current that day. During the tests, although looking at the boat we thought that it was still, the GPS RTK captured a small velocity of about 0.1 m/s. For this reason, the starting velocity of the real boats (dotted blue line) was not zero. These charts show that both simulated and real boats were accurate in terms of velocity variation over the time and also accurate in terms of maximal velocity. The mean absolute error and standard deviation for the airboat are 0.0312 m/s and 0.0481 m/s (respectively), so 94.7% of errors are in the range of one standard deviation. While the mean absolute error and standard deviation for the differential boat are 0.0507 m/s and 0.0742 m/s (respectively), so 96.6% of errors are in the range of one standard deviation.

## 5. Experimental Results

This section demonstrates the resulting effects of our simulator over the four modeled USVs. Section 5.1 and Section 5.2 show two different examples of how winds and water models can affect the trajectory of the boats. Section 5.3 is specially dedicated to evaluating the performance of the sailboat according to the wind speed and direction. Section 5.4 evaluates the four USVs performing three different tasks for sake of trajectory comparison. This section also shows the modified trajectory when wind and water disturbances are enabled in the simulation. Section 5.5 presents the trajectory performance of a USV with a simple controller while it performs a typical post-disaster bridge inspection with fast water current and winds.

### 5.1. Boat at Variable Disturbances

**Figure 8:**
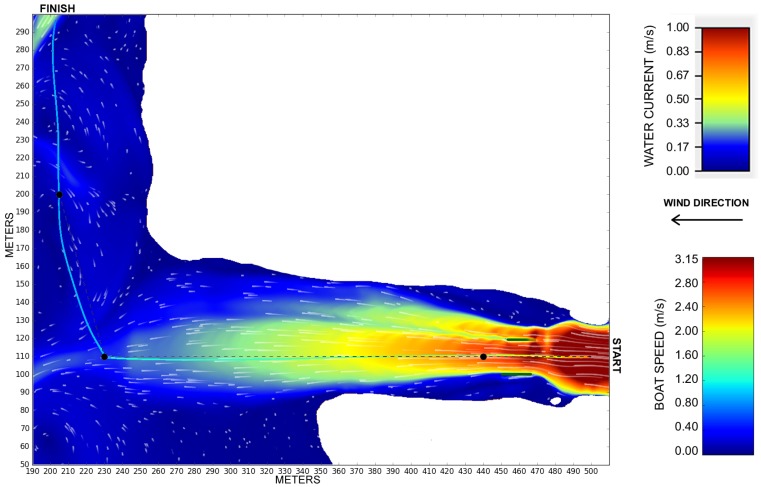
Trajectory of a differential boat in the environment presented in Figure 5. The trajectory color represents the boat speed along the path.

This section describe an experiment showing the differential boat crossing the river with the proposed disturbances, as seen in Figure 5. The figure shows the unique ability of the proposed simulator to disturb the boat’s pose and speed according to a hydrological model of the water body. Figure 8 shows the boat starts at the right-hand side and goes to the top left side of the map. The boat has a higher speed when it is leaving the straight canal, where the water current is faster, and the speed decreases as the boat reaches the open waters on the left-hand side. The total traveled distance is 452.38 m. The boat took 376.98 s (average speed of 1.2 m/s with a peak speed of about 3.04 m/s) to complete the trajectory since it is running in the same direction of the water and wind flows, helping the boat to gain speed.

### 5.2. Different Water Speeds of the River

This section shows that the simulator is coherent with some basic river navigation guidelines. For instance, boats that sail upstream should stay close to the river bank, where the water current is slower than in the middle of the river. On the other hand, boats that sail downstream should stay in the middle, as they can advantage of the river flow, saving fuel.

**Figure 9:**
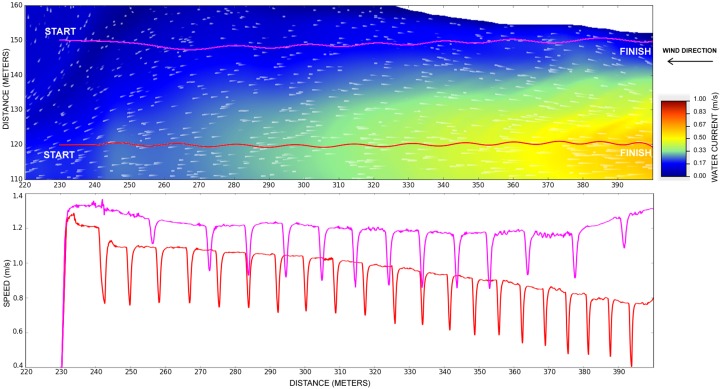
Different navigation speeds of the differential boat going upstream, against the river current.

Figure 9 represents two different trajectories taken by the differential boat. The pink line, close to the river banks, represents a trajectory where the water current is slower. The red line, in the middle of the river, represents a trajectory where the water current is faster. Both trajectories are starting at the left hand side, thus the water current is in the opposing the direction of the boat. The pink trajectory took 144.9 s to reach the destination, with an average speed of about 1.10 m/s. The red trajectory took 187.2 s to completion, resulting in an average speed of about 0.85 m/s.

The second part of Figure 9 also shows the boat speed for both trajectories during the time. It can be seen that the river bank trajectory, represented by the pink line, keeps a fairly constant speed of about 1.1 and 1.3 m/s. However, the middle river trajectory represented by the red line, has a clear speed reduction as the boat gets closer to the area with faster water currents. The speed starts with about 1.2 m/s but it ends with a speed below 0.8 m/s. Both trajectories have sudden reductions in speed. This is an effect of the controller, when it steers the boats. The boat close to river banks presented fewer speed reductions than the boat in the middle, since the water close to riversides presents less turbulence and small speed than on the middle river water.

### 5.3. SailBoat Performance

**Figure 10:**
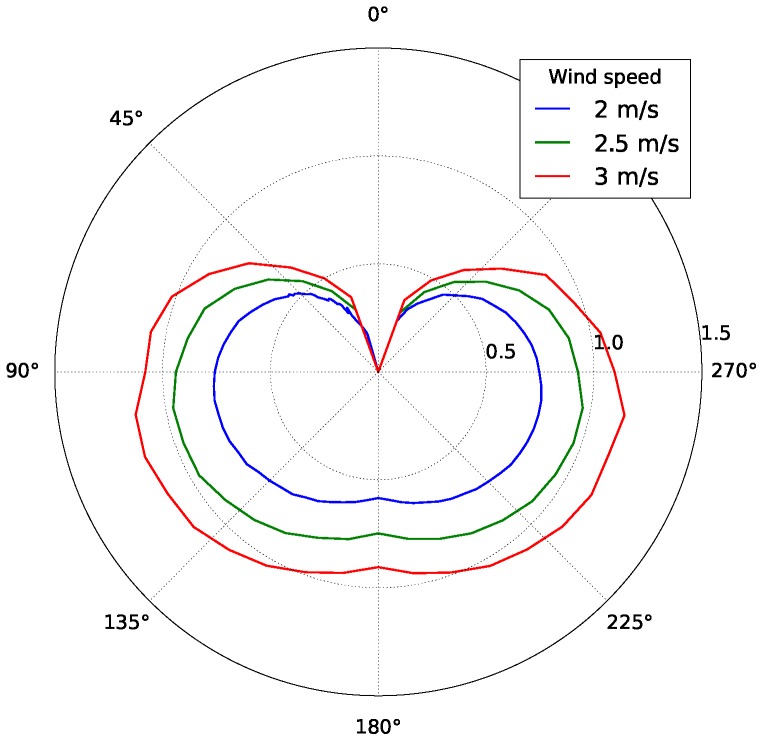
Polar diagram of the sailboat model for a 2 m^2^ sail area.

A particularly interesting model to demonstrate the full features of the proposed simulation is the sailboat because its performance is directly related to accurate disturbance models (wave, winds, water current) [37]. For example, a generic sailboat performance is usually described by a so-called polar graph, similar to the one presented in Figure 10. A sailboat polar diagram is a quick way of visualizing the maximum speed that a sailboat can reach under certain conditions of wind speed and direction. For example, in Figure 10, for a wind with angle 90° and speed of 2 m/s the maximum sailboat speed is about ≈0.75 m/s. This particular polar graph presented in Figure 10 describes the performance of our sailboat model, which incorporates the kinetics of our Foil Dynamics Plugin. It is a typical sailboat profile, where the sailboat velocity is zero when sailing directly upwind (0°) and not optimal when sailing directly downwind 180°. In addition, usually, a sailboat can not gain forward speed if the wind direction is between −30° and 30°. These features of the polar graph demonstrate the overall realism of the simulation for modeling sailboats.

### 5.4. Differential Boat under Disturbances

**Figure 11:**
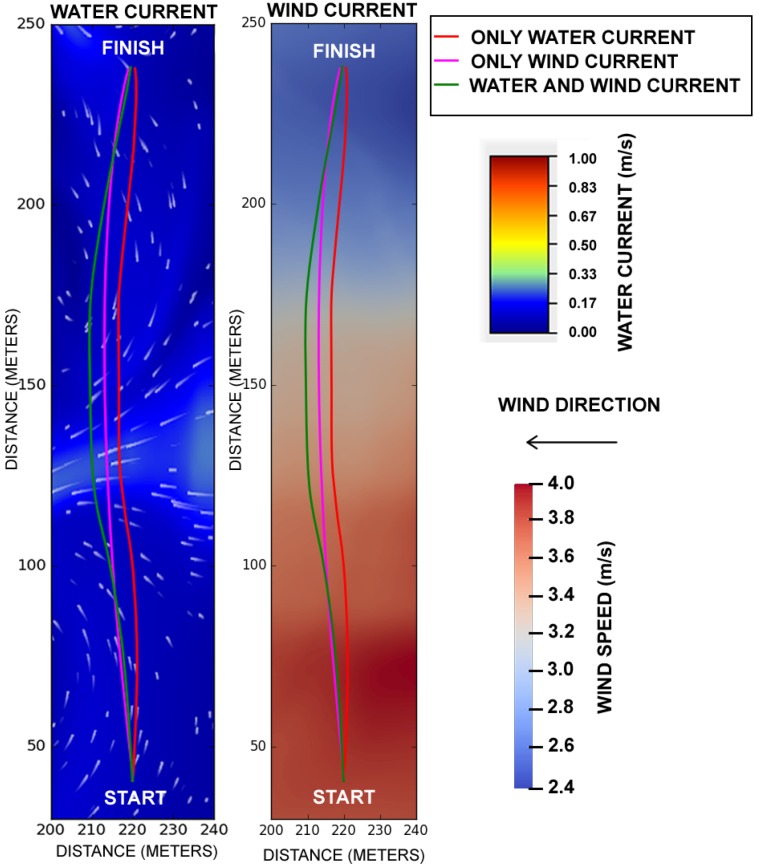
Differential boat travelling on straight line. The red line presents the trajectory of the boat when it is affected only by the water current. The pink trajectory, when the differential boat is affected only by the wind current. The green trajectory when the boat is affected simultaneously by wind and water current.

Figure 11 shows how the resulting trajectory of the differential boat under different disturbances. If all disturbances are turned off, then the boat would follow a perfect straight line to the destination. The image shows the trajectory with only water current (red line), wind current (pink line), and both currents simultaneously (green line). One can see that the trajectory is more distorted when both disturbances are on, and nearby the place where the water current is faster. The turbulence of the water stream disturbs the movement of the boat in a variable way along the course, which is not observed in other USV simulation models.

### 5.5. Bridge Inspection

Bridge inspection is an important task to be executed both before a disaster, as a disaster mitigation action, or after the disaster, as a response action to check if a bridge can be used after a hurricane. The issue is that, for instance, right after a disaster such as a hurricane or a flood, the weather condition might still not be safe for perform manned inspections, putting first responders at risk of a potentially collapsing bridge subject to high water currents, waves, and strong winds.

Due to these risks, some researchers proposed to use unmanned vehicles for this kind of inspection, including the use of USVs [38,39]. The main problem is that these research vehicles were not thoroughly tested under such harsh environments, especially considering that every disaster scenario is unique, with its own complexities. Moreover, testing a real USV in a disaster-like scenario puts the USV itself at risk, since while it is still being tested, it is expected to have some failures. A failure, in this case, might cause collision, damage or even loss of the entire USV.

**Figure 12:**
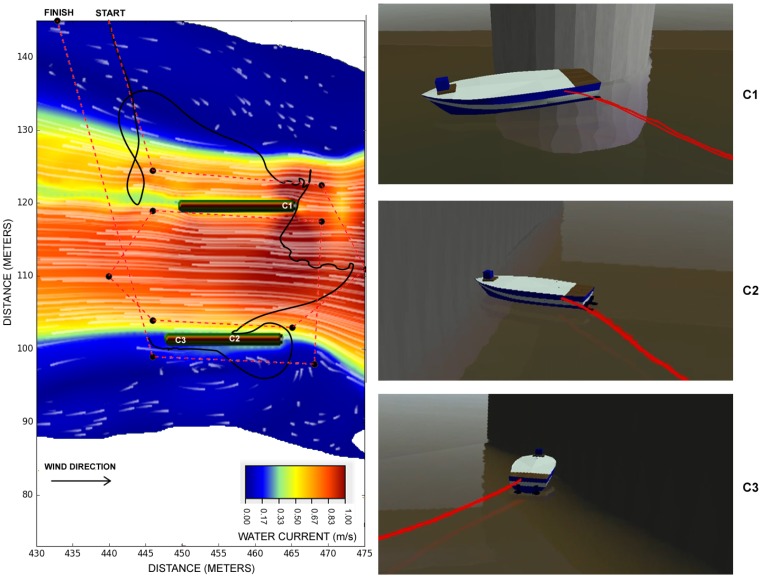
Bridge inspection scenario with differential boat.

Considering the complex task of testing a USV for a harsh scenario such as an after-disaster bridge inspection, we propose a simulation environment for bridge inspection. Figure 12 is a detailed view of the same bridge presented in Figure 6, located at 30°02′50.5″ S 51°13′57.7″ W, in the city of Porto Alegre, Brazil, nearby the Dilúvio’s river mouth. In this figure we can see that the water current is faster in between the two pillars of the bridge, and the current is slower by the river banks. The differential boat started in the top position of the image and it has the goal of capturing images in the positions marked with dots. The black solid line represents the trajectory executed by the USV. The dashed line represents the expected trajectory. The points with C1, C2, and C3 marks show places of collision with the pillars, detailed in the screenshots.

One of the reasons for the poor navigation performance of the USV is related to its controller design. As mentioned before, all four boat models use the same orientation-based control strategy, where the goal is to keep the USV pointing to the next waypoint. However, in situations like this bridge inspection scenario, the Differential boat controller is expected to have a poor performance since it does not take water current and wind into account. It could, for instance, use the areas of slower current to make maneuvers and go up the river. Then it could enter into the fast current up river (right-hand side of the image), to navigate with the current. This example shows one usage of the proposed simulator, which is to test the USV under harsh environments, testing and benchmarking different strategies to complete the tasks assigned to the USV.

## 6. Conclusions

This paper presented an open source simulator that models waves, wind, and water currents. The proposed buoyancy effect, more realistic than those of previous simulators, makes waves induce roll and pitch movements on the boat. The proposed wind module applies a force on the boat according to the existent wind obstacles in the scenario, and also according to the boat’s shape above the water. The water current applies variable forces on the boat, using a *d*e facto standard hydrological simulator. Previous simulators, on the other hand, modeled both wind and water current as a single constant applied to the entire scenario. Other contributions of this paper include the four different ready-to-use USV models and a model of a real scenario for evaluation of the boat’s performance under varying degrees of disturbances.

The results corroborate the importance to model these environmental disturbances since the trajectory discrepancies increase according to the degree of disturbances. Multiple scenarios and tasks are required to assess a controller performance since the results can vary significantly among these scenarios. Finally, we expect that this simulator can be used by the USV research community to design new navigation strategies under harsh environmental conditions usually present after a disaster scenario such as hurricane and floods.

In the future, we will devise a strategy to make boats affect the waves, creating splash waves and, consequently, making such disturbances influence nearby boats. This feature is important when simulating multiple boats sharing the same scenario. We will also continue to test different validation strategies for the disturbances models.

## Figures and Tables

**Table 1:** Physical fidelity of multiple Unmanned Surface Vehicle simulators.

Simulator	Waves	Buoyancy	Water Currents	Wind Currents	Thruster Underwater	Thruster above Water	Foil
**UWSim**	√	√	×	×	√	×	×
**Gazebo**	×	×	×	×	√√	√√	×
**Freefloating Gazebo**	√	√	√	×	√√	√√	×
**VREP**	√	√	×	×	√	√√	×
**RobotX Simulator**	√	√√	×	√	√√	√√	×
**USVSim**	√√	√√	√√	√√	√√	√√	√√

**Table 2:** Lutra Airboat and Lutra Prop parameters.

Parameter	Lutra Airboat	Lutra Prop
Length	106 cm	106 cm
Width	48 cm	48 cm
Height	45 cm	15 cm
Hull Volume	∼0.02 m^3^	∼0.02 m^3^
Weight	9 Kg	9.7 Kg
Extra Payload	3 Kg	3 Kg
Thruster Force	3.1 N	22.54 N
Linear drag	6.9	11.33
Maximum speed	0.67 m/s	1.41 m/s

**Table 3:** Propulsion forces of the boats. PWM stands for Pulse Width Modulation.

Lutra Airboat			Lutra Prop	
PWM	Force		PWM	Force
2000	3.13 N		1800	-
1850	2.64 N		1700	22.54 N
1750	2.35 N		1600	17.93 N
1650	1.86 N		1553	16.46 N
1500	0		1500	0

## Abbreviations

The following abbreviations are used in this manuscript:

3D	Three Dimensional
CFD	Computational Fluid Dynamics
GNC	Guidance Navigation and Control
GPS	Global Positioning System
HDF	Hierarchical Data Format
NTRIP	Networked Transport of RTCM via Internet Protocol
PWM	Pulse Width Modulation
RTK	Real Time Kinematic
USV	Unmanned Surface Vehicle
UUV	Unmanned Underwater Vehicle
UGV	Unmanned Ground Vehicle
